# Crosstalk between G protein-coupled receptors (GPCRs) and tyrosine kinase receptor (TXR) in the heart after morphine withdrawal

**DOI:** 10.3389/fphar.2013.00164

**Published:** 2013-12-27

**Authors:** Pilar Almela, Juan-Antonio García-Carmona, Elena Martínez-Laorden, María-Victoria Milanés, María-Luisa Laorden

**Affiliations:** Department of Pharmacology, Faculty of Medicine, University of MurciaMurcia, Spain

**Keywords:** morphine withdrawal, PKA, MAPK, heart, tyrosine hydroxylase, HA-1004

## Abstract

G protein-coupled receptors (GPCRs) comprise a large family of membrane receptors involved in signal transduction. These receptors are linked to a variety of physiological and biological processes such as regulation of neurotransmission, growth, and cell differentiation among others. Some of the effects of GPCRs are known to be mediated by the activation of mitogen-activated extracellular kinase (MAPK) pathways. Cross-talk among various signal pathways plays an important role in activation of intracellular and intranuclear signal transduction cascades. Naloxone-induced morphine withdrawal leads to an up-regulation of adenyl cyclase-mediated signaling, resulting in high expression of protein kinase (PK) A. In addition, there is also an increased expression of extracellular signal regulated kinase (ERK), one member of MAPK. For this reason, the crosstalk between these GPCRs and receptors with tyrosine kinase activity (TKR) can be considered a possible mechanism for adaptive changes that occurs after morphine withdrawal. Morphine withdrawal activates ERK1/2 and phosphorylated tyrosine hydroxylase (TH) at Ser31 in the right and left ventricle. When N-(2-guanidinoethyl)-5-isoquinolinesulfonamide (HA-1004), a PKA inhibitor was infused, the ability of morphine withdrawal to activate ERK, which phosphorylates TH at Ser31, was reduced. The present finding demonstrated that the enhancement of ERK1/2 expression and the phosphorylation state of TH at Ser31 during morphine withdrawal are dependent on PKA and suggest cross-talk between PKA and ERK1/2 transduction pathway mediating morphine withdrawal-induced activation of TH. Increasing understanding of the mechanisms that interconnect the two pathway regulated by GPCRs and TKRs may facilitate the design of new therapeutic strategies.

## Introduction

The development of opioid addiction involves complex adaptive changes in opioid receptors and associated signaling systems leading to neuronal plasticity in specific brain regions (Nestler and Aghajanian, 1997; Ueda, 2004). In addition to the neurobehavioral consequences of opioid addiction, there is a strong association between drug addiction and cardiac disorders. There are studies in the literature supporting mainly the effect of cocaine abuse (Aquaro et al., 2011; Basso et al., 2011) but less in heroin abuse. However, various types of cardiac arrhythmias have been described in heroin addicts. Moreover, street heroin addicts frequently die suddenly, and there is evidence that this is an arrhythmia-related event (Nerantzis et al., 2011). Despite the clinical relevance of an association between addiction and cardiovascular disorders, little is known about the pathophysiology or mechanisms underlying this comorbidity. The majority of cardiology studies were oriented on clinical usage of this drug and current cardiovascular research has been limited to the evaluation of factors or pathways believed to contribute to its physiological actions (Jiang et al., 2006; Xu et al., 2011). So, investigation about the mechanisms implicated in the cardiac adaptive changes that occur during morphine withdrawal deserves more attention.

Although the μ opioid receptor is negatively coupled to the adenylate cyclase/cAMP-dependent protein kinase (PK) A pathway upon acute stimulation (Childers, 1991), the pathway is up-regulated in cardiac tissues after chronic morphine treatment (Milanés et al., 1999). Furthermore, it has been demonstrated that PKA plays an important role in regulating protein phosphorylation and contraction in cardiac muscle [see Sugden and Bogoyevitch, (1995) for review]. Cardiac inotropic activity is strongly regulated by intracellular PKA (Hussain et al., 1999; Kamp and Hell, 2000). Previous studies in our laboratory have demonstrated that naloxone administration to morphine-dependent rats leads to an increase in the force and rate of contraction in different cardiac tissues (Rabadán et al., 1997, 1998). In addition, it has been demonstrated that withdrawal from morphine is associated with a marked increase in the ventricular levels of cAMP in parallel with an enhancement of noradrenaline (NA) turnover (Milanés et al., 2000).

Extracellular signal-regulated kinase (ERK), one member of mitogen-activated extracellular kinase (MAPK) family, transduces a broad range of extracellular stimuli into diverse intracellular responses. ERK signaling pathway could be important as regulator of cardiac function [see Michel et al. 2001 for review]. Recently, several studies have shown that this pathway contributes to naloxone-precipitated withdrawal in morphine dependent rats (Ren et al., 2004; Almela et al., 2007a, 2008).

It is now appreciated that crosstalk among various signal pathways plays an important role in activation of intracellular and intranuclear signal transduction cascades. Different studies have shown a crosstalk between cAMP and MAPK (see Stork and Schmitt, 2002, for reviews). cAMP stimulates MAPK activity in cultured neurons (Villalba et al., 1997; Vossler et al., 1997) and is required for its nuclear translocation (Impey et al., 1998). Crosstalk between cAMP/PKA and MAPK pathways is necessary to regulate genetic expression (Sengupta et al., 2007). It has been demonstrated that PKA, MAPK, and mitogen- and stress-activated protein kinase (MSK1) are activated in the same subset of CA1 pyramidal neurons, and that Ca^2+^-stimulated adenyl cyclase activity is indispensable for the training-induced activation of MAPK, MSK1, and CREB (cAMP response element binding protein) (Sindreu et al., 2007). Above mentioned studies showed that G protein-coupled receptors (GPCRs) and receptors with tyrosine kinase activity (TKR) represent distinct and linear signaling units that converge on down-stream targets. Therefore, the increase in PKA activity may be necessary to support the activation of MAPK during morphine withdrawal in the heart. However, there is no evidence that PKA activation is required for stimulation of ERKs and subsequently phosphorylation of TH at Ser 31 in morphine-dependent rats. The present study examine whether the interaction between PKA and ERK signaling pathways, in the heart, mediates the enhancement of TH phosphorylation observed after naloxone administration to morphine-dependent rats.

## Methods

Male Sprague-Dawley rats (220–240 g at the beginning of the experiments) were housed four-to-five per cage under a 12-h light/dark cycle (light: 8:00–20:00 h) in a room with controlled temperature (22 ± 2°C), humidity (50 ± 10%), food and water available *ad libitum* and handled for several days preceding the experiment to minimize stress, as previously described (Laorden et al., 2000). All surgical and experimental procedures were performed in accordance with the European Communities Council Directive of 24 November 1986 (86/609/EEC) and the local Committee.

### Experimental procedure

Rats were rendered tolerant/dependent on morphine by s.c. implantation of morphine base pellets (75 mg), one on day 1, two on day 3, and three on day 5, under light ether anaesthesia (Rabadán et al., 1997; Milanés et al., 2000). Control animals were implanted with placebo pellets containing lactose instead of morphine, on the same time schedule. This procedure has been shown to produce consistent plasma morphine concentrations beginning a few hours after the implantation of the pellets and a full withdrawal syndrome after acute injection of opioids antagonist (Frenois et al., 2002). Dependence on morphine remained constant for 15 days (Gold et al., 1994). On day 8, the animals treated with morphine or placebo pellets were injected with saline s.c. or naloxone (2 mg/kg s.c.). We used this model because the adaptive changes observed in the heart are more evident after naloxone-precipitated withdrawal than after deprivation from morphine.

The weight gain of the rats was checked along the treatment to ensure that the morphine was liberated correctly from the pellets because it is known that chronic morphine treatment induces a decrease in body weight gain due to lower caloric intake. In addition, body weight loss was determined as the difference between the weight checked immediately before saline or naloxone injection and a second determination made 60 min later.

In order to determine the effects of PKA and PKC on the morphine withdrawal-induced changes in ERK1/2, animals were continuously infused for 7 days, via s.c. osmotic minipumps (Alzet mod. 2001, which deliver at 1 μL/h; Alza, Palo Alto, CA, USA), with HA-1004, a PKA selective inhibitor (Hidaka et al., 1984) (40 nmol/day), calphostin C, a PKC selective inhibitor (Kobayashi et al., 1989) (40 pmol/day), or vehicle. PKA inhibitor was dissolved in sterile water and PKC inhibitor in dymethylsulphoxide (DMSO) and serially diluted in MiIliQ-water (final concentration of DMSO was 0.06%). Minipumps were implanted simultaneously with the chronic morphine or placebo pellets. Pumps were primed for 5 h before implantation at 37°C in sterile saline in order to obtain an optimal flow rate (1 μL/h). On day 8, morphine withdrawal syndrome was induced by s.c. naloxone (2 mg/kg) injection. To determine the role of ERK in TH phosphorylation in the heart, TH phosphorylated at Ser31 levels were determined in morphine dependent and control rats treated, 1 h before the injection of naloxone or saline, with SL327, a selective inhibitor of mitogen-activated protein kinase (MAPK)/ERK kinase (MEK) (Atkins et al., 1998). This inhibitor was dissolved in DMSO (100%) and injected intraperitoneally at an injection volume of 1 ml/kg at dose of 100 mg/kg (Almela et al., 2007a). Other groups of rats were treated with HA-1004 to determine the role of PKA in ERK and TH phosphorylation.

Animals were killed by decapitation 60 or 90 min after naloxone or saline administration in order to analyze ERKs and TH phosphorylation. The hearts were rapidly removed, and the right and left ventricle were dissected, fresh-frozen, and stored immediately at −80°C until use.

### Western blot analysis

Samples were placed in homogenization buffer phosphate buffered saline, 2% sodium dodecylsulfate (SDS) plus protease (Boehringer Mannhein, Germany) and phosphatase inhibitor Cocktail Set (Calbiochem, Germany), and homogenized for 50 s prior to centrifugation at 6000 g for 20 min at 4°C. Total protein concentrations were determined spectrophotometrically using the bicinchoninic acid method (Wiechelman et al., 1988). The optimal amount of protein to be loaded was determined in preliminary experiments by loading gels with increasing protein contents (25–100 μg) from samples of each experimental group. Equal amounts of protein (50 μg/lane) from each sample were loaded on a 10% SDS-polyacrilamide gel (SDS-PAGE), electrophoresed, and transferred onto poly vinylidene difluoride (PVDF) membrane using a Mini Trans-Blot Electrophoresis Transfer Cell (Bio-Rad Laboratory, CA, USA). Non-specific binding of antibodies was prevented by incubating membranes in 1% bovine serum albumin (BSA) in tris buffer saline tween (TBST: 10 mM Tris-HCl, pH 7.6, 150 mM NaCl, 0.05% Tween 20). The blots were incubated overnight at room temperature (for pTH) or at 4°C (for pERK, PKA, PKC δ), with the following primary antibodies: specific polyclonal PKA catalytic subunit antibody (1:2000 dilution; sc-903, Santa Cruz Biotechnology, Santa Cruz, CA, USA); polyclonal anti PKCδ (1:1000 dilution; p8333, Sigma Chemical Co., ST Louis, MO, USA); monoclonal anti-pERK1/2 (1:1000 dilution; sc-7383, Santa Cruz Biotechnology, Santa Cruz, CA), polyclonal anti-pSer31 TH (1:250 dilution; AB5423, Chemicon International, CA, USA), in TBST with BSA. After extensive washings with TBST, the membranes were incubated for 1 h, at room temperature, with peroxidase-labeled secondary antibodies (anti-rabbit sc-2004 for PKA, PKCδ, pTH, total-ERK, Santa Cruz; anti-mouse sc-2005 for phospho-ERK1/2, Santa Cruz) both at 1:5000 dilution. After washing, immunoreactivity was detected with an enhanced chemiluminescence western blot detection system (ECL, Amersham-Pharmacia-Biotechnology, Madrid, Spain) and visualized by Amersham Hyperfilm-ECL. Quantification of PKA (42 kDA), PKCδ (78 kDA), phospho-ERK1/2 (42 and 44 kDA) and TH phosphorylated at Ser31 (60 kDA) bands was carried out by densitometry (AlphaImager, Nucliber, Madrid, Spain). The integrated optical density of bands was normalized to the background values. The optical density of the bands was normalized as a percentage of average of control. Relative variations between bands of experimental samples and control samples were calculated in the same image. We used β-actin or total-ERK as our loading control for all the experiments. Before reprobing, blots were stripped by incubation with stripping buffer (glycine 25 mM and SDS 1%) pH2, for 1 h at 37°C. Blots were subsequently reblocked and probed with 1:1000 anti-β actin (Cell Signaling, 43 kDA) or anti-total ERK (sc-154, Santa Cruz Biotechnology, Santa Cruz, CA, USA). The ratio of PKA/β-actin, PKCδ/β-actin, phospho-ERK1/total ERK, phospho-ERK2/total ERK and phosphoTH/β-actin was plotted and analyzed.

### Hemodynamic parameters

The rats were anesthetized with thiopental sodium (40 mg/kg, i.p.), intubated and placed on a heated table to maintain body temperature at 37°C. A polyethylene cannula (PE-50) was placed in the right femoral artery to measure blood pressure and heart rate (HR). Catheters were connected to pressure transducers (L969-A07 Abbott Ireland, Sligo, Ireland), monitored on a PowerLab8/30 (ADInstruments, Pty Ltd., Oxford, UK) and analyzed with LabChart software (ADInstruments, Pty Ltd., Oxford, UK). Naloxone (2 mg/kg) was injected subcutaneously after a 30 min stabilization period and its effect on mean arterial blood pressure (MAP) and HR was evaluated in rats implanted with placebo or morphine concomitantly treated with vehicle or HA-1004.

### Immunohistochemistry

Rats were killed with an overdose of pentobarbital (100 mg/kg, i.p.) for phospho-ERK1/2 determination 90 min after naloxone or saline administration. Following anaesthesia, rats were perfused through the descending aorta with 300 ml of PBS (pH 7.4) followed by 500 ml of cold, 4% paraformaldehyde in PBS. Following perfusion, hearts were removed, postfixed in the same fixative and stored at 4°C overnight. The samples were then processed routinely and embedded in paraffin wax. The sections (5 mm thick) were obtained on a microtome (HM310, Microm, Barcelona, Spain) and were immunostained. The sections were deparaffinised in xylene and dehydrated in a graded ethanol series. Endogenous peroxidase activity was destroyed by 30 min treatment with 0.3% hydrogen peroxide in PBS. Sections were washed in three 5 min intervals of PBS and treated with NSS-PBS (PBS containing 1% normal swine serum; Dako, Glostrup, Denmark; and 0.5 Triton X-100) for 30 min. Anti-phospho-ERK1/2 antibody (diluted 1:1500 in NSS-PBS, overnight) was used as a primary antibody and horse antimouse IgG (1:400, 1 h; Vector) as a secondary antibody. The bound primary antibody was localized by biotinylated secondary anti-rabbit IgG (diluted 1:200 in NSS-PBS; Vector, Burlingame, CA, USA) and subsequently with the avidin–biotin complex (ABC kits; Vector) at room temperature for 1 h each. Visualization of antigen-antibody reaction sites used 0.033% 30, 30-diaminobenzidine (DAB; Sigma) and 0.014% H2O2 in 0.05 M Tris-HCl buffer for 7 min. The reaction was stopped in PBS and slides were coverslipped with DPX.

### Drugs and chemicals

Pellets of morphine base (Alcaliber Laboratories, Madrid, Spain) or lactose were prepared by the Department of Pharmacy and Pharmaceutic Technology (School of Pharmacy, Granada, Spain); sodium dodecylsulphate, polyacrylamide gel and PVDF membranes were obtained from Bio-Rad Laboratory (Teknovas, Bilbao, Spain). Naloxone HCl and western blot reagents were purchased from Sigma Chemical Co. (St Louis, MO, USA). Naloxone HCl was dissolved in sterile 0.9% NaCl (saline) and administered in volumes of 0.1 mL/100 g body weight. HA-1004 [N(-2′ guanidinoethyl-5-isoquinolinesulfonamide)] was purchased from Sigma Chemical Co. and dissolved in Milli-Q (Millipore, Bedford, MA, USA) sterile water. Calphostin C (2-(12-(2-(benzoyloxy)propyl)-3,10-dihydro-4,9-dihydroxy-2,6,7,11-tetrametoxy-3,10-dioxo-1-perylenyl)-1menthylethyl carbonic acid 4-hydroxyphenyl ester) was purchased from RBI (Natick, MA, USA). The chronic delivery of HA-1004 or Calphostin C was achieved by means of Alzet 2001 osmotic minipumps (Alza, Palo Alto, CA), which deliver at 1 μL/h. SL327 alpha-[Amino-(4-aminophenylthio)methylene]-2-(trifluoromethyl) benzeneacetonitrile was purchased from Ascent, Scientific, Bristol, UK.

### Statistical analysis

The results are expressed as the mean ± s.e.m. Data were analyzed by One Way analysis of variance (ANOVA) followed by the Newman–Keuls *post-hoc* test. Body weight gain and loss in naïve- and morphine-dependent rats was analyzed by unpaired Student's *t*-test. One Way ANOVA followed by Dunett's multiple comparison test was used when required. Differences with a *p* value less than 0.05 were considered significant.

## Results

Before performing the experiments, we assessed the efficacy of chronic treatment with morphine by pellets implantation, which has been previously shown to induce tolerance and dependence to the effects of morphine (González-Cuello et al., 2004).

### Changes in body weight

**Figure 1:**
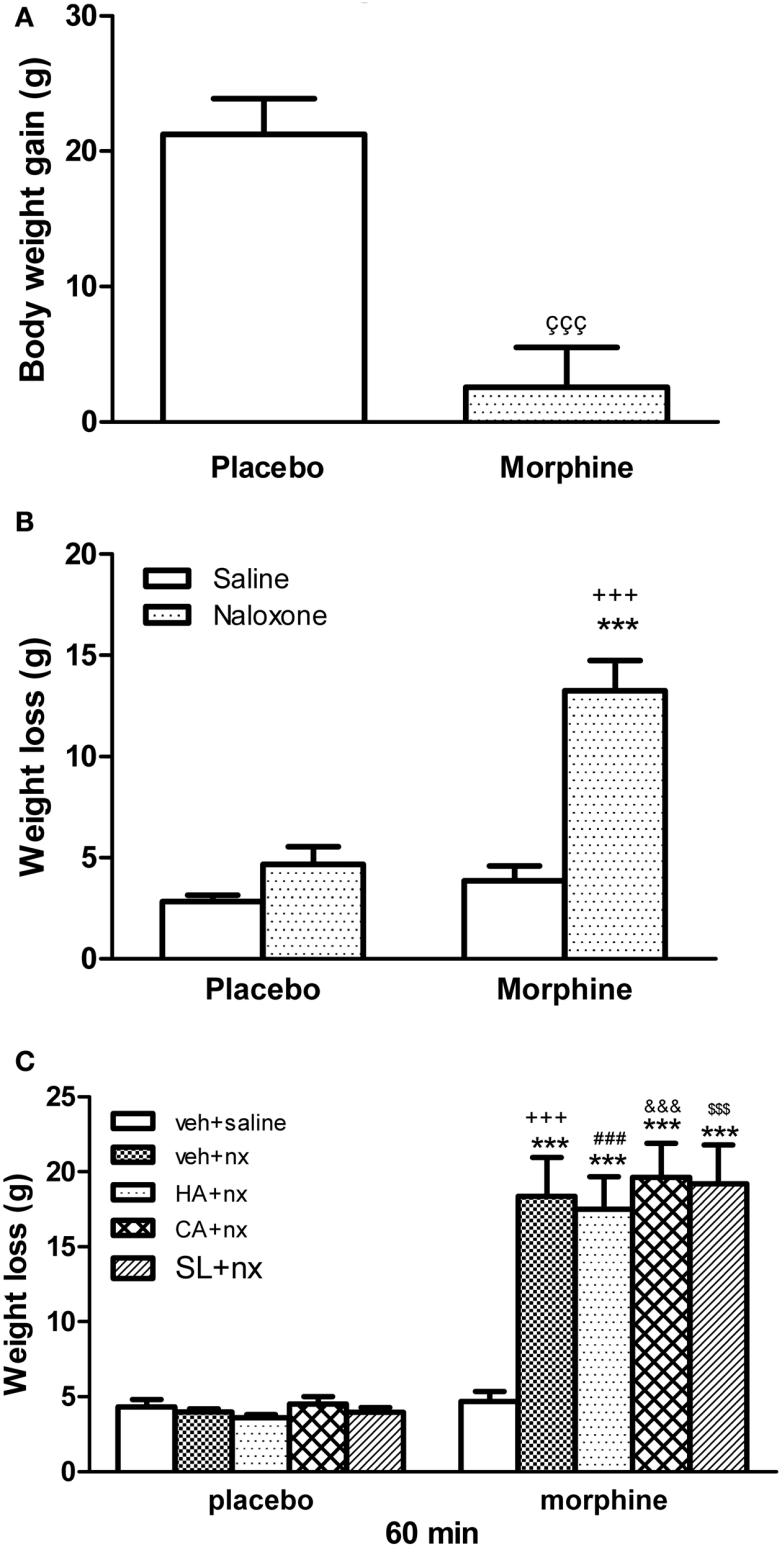
**(A)** Effect or morphine pellet-implantation on body weight gain. **(B)** Body weight loss after naloxone-precipitated withdrawal. Animals received subcutaneous implantation of placebo or morphine pellets for 7 days. On day 8, rats were injected with saline or naloxone (nx, 2 mg/kg) and decapitated 60 min later. **(C)** Other groups of animals were pretreated with vehicle (veh), HA-1004 (HA, 40 nmol/day), calphostin C (CA, 40 pmol/day) or SL-327 (SL, 100 mg/kg i.p.). Data are the mean ± s.e.m. ^ççç^*p* < 0.001 vs. placebo; ^***^p < 0.001 vs. morphine-dependent group receiving saline instead of naloxone; ^+++^*p* < 0.001 vs. the control group injected with naloxone; ^###^*p* < 0.001 vs. placebo+HA+nx; ^&&&^*p* < 0.01 vs. placebo+CA+nx; ^$$$^*p* < 0.001 vs. placebo+SL+nx.

The weight of the animals was recorded since the day of pellets implantation until the day of sacrifice (day 8), before receiving any injection. Rats treated with morphine showed a significantly lower body weight gain than animals receiving placebo pellets (Figure 1A). Chronic morphine-treated animals showed a significant weight loss 60 after naloxone injection when compared with placebo-pelleted group also receiving naloxone (Figure 1B). The injection of naloxone in rats chronically treated with HA-1004, Calphostin C or SL327 concomitantly with morphine induced a weight loss, similar to the group chronically pretreated with vehicle plus morphine (Figure 1C). Morphine-withdrawn animals treated with vehicle, HA-1004, calfostin C or SL327 displayed characteristic abstinence symptoms: Wet-dog shakes, teeth chattering, ptosis, tremor, piloerection, lacrimation, rhinorrhea, chromodacryorrhea and spontaneous jumping.

### PKA and PKC δ expression in the right ventricle

**Figure 2:**
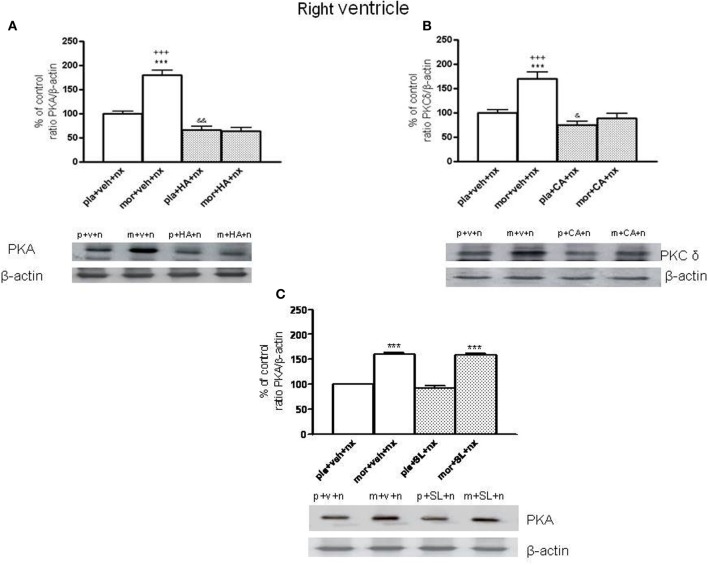
**Inmunoblots of PKA (A) and PKCδ (B) in right ventricle from placebo (pla, p)- or morphine (mor, m)-dependent rats after naloxone-precipitated withdrawal in vehicle (veh, v)-infused rats and in animals chronically administered with HA-1004 (HA) or Calphostin C (CA)**. Animals received subcutaneous implantation of placebo or morphine (75 mg) pellets for 7 days and concomitantly were infused with vehicle, HA or CA. On day 8, rats were decapitated 90 min after naloxone (nx, n.) administration in presence of vehicle, HA, CA or SL327 (SL) **(C)**. SL was administered 1 h before naloxone injection. The immunoreactivity corresponding to PKA or PKCδ is expressed as a percentage respect to the control group (pla+veh+nx; defined as 100% value). Data are the mean ± s.e.m. (*n* = 4–5). ^***^p < 0.001 vs. pla+veh+nx or pla+SL+nx; ^+++^*p* < 0.001 vs. the group treated with mor+HA+nx or mor+CA+nx; ^&^*p* < 0.05, ^&&^*p* < 0.01 vs. pla+veh+nx. Bottom panels: representative bands from autoradiograms at the known apparent molecular weight for PKA and PKCδ.

In this study, we have evaluated PKA or PKC δ levels in the right ventricle blocking PKA activity using HA-1004 or SL327 or the inhibition of PKC δ by calphostin C. As shown in Figure 2A, chronic pre-treatment with HA-1004 concomitantly with morphine antagonized the expression of PKA in both controls and morphine-withdrawn animals. However, the administration of SL327 did not inhibit the increase in PKA expression observed after naloxone administration to morphine-dependent animals (Figure 2C). Regarding calphostin C, this inhibitor blocked the expression of PKC δ observed after naloxone-induced withdrawal in both control and morphine-dependent animals (Figure 2B). Similar results were obtained in the left ventricle. These experiments demonstrated that the doses of HA-1004 and calphostin C used in this study are useful to inhibit the expression of PKA or PKC δ, one of the main PKC isoforms involved in the adaptive changes observed in the heart during morphine withdrawal (Cerezo et al., 2005).

### Effects of HA-1004 and calphostin C on ERK phosphorylation in the heart

**Figure 3:**
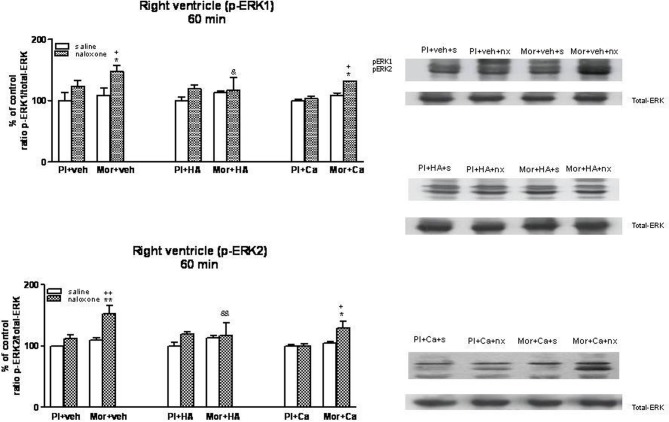
**Western blotting analysis of phospho (p)-ERK1 and phospho (p)-ERK2 immunoreactivity levels in the right ventricle 60 min after saline (s) or naloxone (nx) administration to placebo (Pl)- or morphine (Mor)-treated rats receiving vehicle (veh), HA1004 (HA) or calphostin (Ca)**. The immunoreactivity corresponding to ERK1 or ERK2 is expressed as a percentage respect to the control group (Pl+veh+saline; defined as 100% value). Data are the mean ± s.e.m. (*n* = 4–6). ^*^p < 0.05, ^**^p < 0.01 vs. the morphine dependent group receiving saline instead of naloxone; ^+^*p* < 0.05, ^++^*p* < 0.01 vs. the group pretreated with placebo instead of morphine injected with naloxone; ^&^*p* < 0.05, ^&&^*p* < 0.01 vs. the group receiving vehicle instead of HA. Right panels: representative bands from autoradiograms at the known apparent molecular weight for p-ERK1 or p-ERK2.

**Figure 4:**
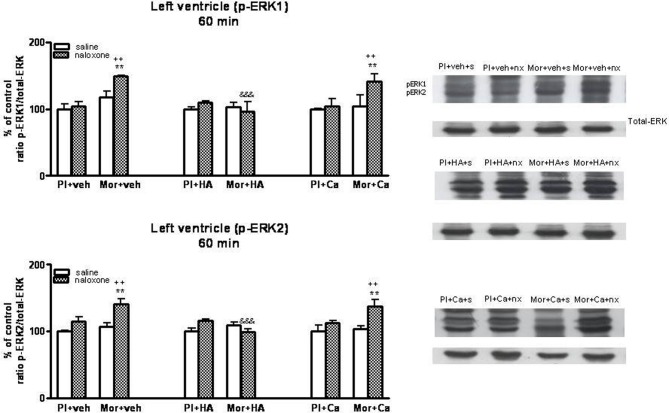
**Western blotting analysis of phospho (p)-ERK1 and phospho (p)-ERK2 immunoreactivity levels in the left ventricle 60 min after saline (s) or naloxone (nx) administration to placebo (Pl)- or morphine (Mor)-treated rats receiving vehicle (veh), HA1004 (HA) or calphostin (Ca)**. The immunoreactivity corresponding to ERK1 or ERK2 is expressed as a percentage respect to the control group (Pl+veh+saline; defined as 100% value). Data are the mean ± s.e.m. (*n* = 4–6). ^**^p < 0.01 vs. the morphine dependent group receiving saline instead of naloxone; ^++^*p* < 0.01 vs. the group pre-treated with placebo instead of morphine injected with naloxone; ^&&&^*p* < 0.001 vs. the group receiving vehicle instead of HA. Right panels: representative bands from autoradiograms at the known apparent molecular weight for p-ERK1 or p-ERK2.

**Figure 5:**
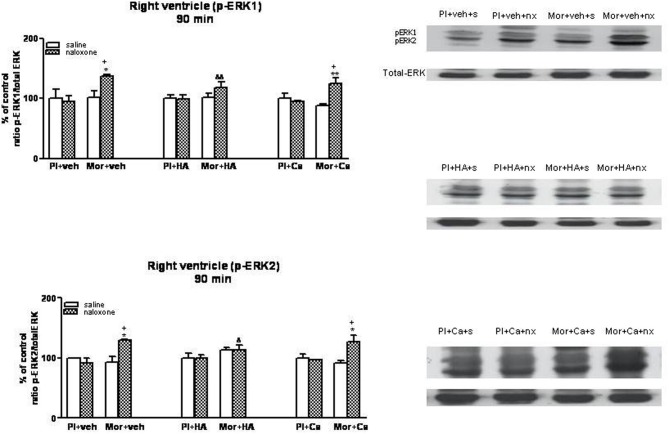
**Western blotting analysis of phospho (p)-ERK1 and phospho (p)-ERK2 immunoreactivity levels in the right ventricle 90 min after saline (s) or naloxone (nx) administration to placebo (Pl)- or morphine (Mor)-treated rats receiving vehicle (veh), HA1004 (HA) or calphostin (Ca)**. The immunoreactivity corresponding to ERK1 or ERK2 is expressed as a percentage respect to the control group (Pl+veh+saline; defined as 100% value). Data are the mean ± s.e.m. (*n* = 4–6). ^*^p < 0.05, ^**^p < 0.01 vs. the dependent group receiving saline instead of naloxone; ^+^*p* < 0.05 vs. the group pretreated with placebo instead of morphine injected with naloxone; ^&^*p* < 0.05, ^&&^*p* < 0.01 vs. the group receiving vehicle instead of HA. Right panels: representative bands from autoradiograms at the known apparent molecular weight for p-ERK1 or p-ERK2.

**Figure 6:**
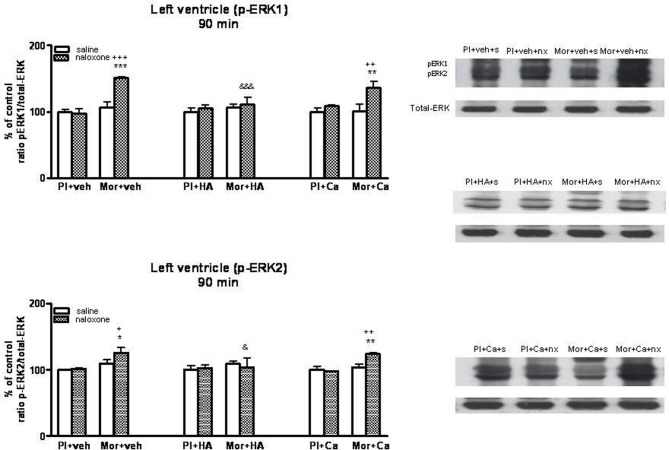
**Western blotting analysis of phospho (p)-ERK1 and phospho (p)-ERK2 immunoreactivity levels in the left ventricle 90 min after saline (s) or naloxone (nx) administration to placebo (Pl)- or morphine (Mor)-treated rats receiving vehicle (veh), HA1004 (HA) or calphostin (Ca)**. The immunoreactivity corresponding to ERK1 or ERK2 is expressed as a percentage respect to the control group (Pl+veh+saline; defined as 100% value). Data are the mean ± s.e.m. (*n* = 4–6). ^*^p < 0.05, ^**^p < 0.01, ^***^p < 0.001 vs. the morphine dependent group receiving saline instead of naloxone; ^+^*p* < 0.05, ^++^*p* < 0.01, ^+++^*p* < 0.001 vs. the group pre-treated with placebo instead of morphine injected with naloxone; ^&^*p* < 0.05, ^&&&^*p* < 0.001 vs. the group receiving vehicle instead of HA. Right panels: representative bands from autoradiograms at the known apparent molecular weight for p-ERK1 or p-ERK2.

Once, we have established the ability of HA-1004 and Calphostin C to antagonize the expression of PKA and PKC observed during morphine withdrawal, then we determined if the increase of PKA or PKC activity was responsible and related with the increase of ERK phosphorylation showed in withdrawn rats. For this purpose, the selective PKA inhibitor HA-1004 or the PKC inhibitor Calphostin C were co-administered with morphine. At different time point, chronic treatment with HA-1004, concomitantly with morphine, antagonized the increase in the ERK1/2 phosphorylation observed during morphine withdrawal in the right (Figures 3, 5) and left ventricle (Figures 4, 6). Calphostin C did not prevent the increase in ERK1/2 observed in the right ventricle or left ventricle 60 (Figures 3, 4) or 90 min (Figures 5, 6) after the injection of naloxone to morphine-dependent rats. These results suggest a crosstalk between PKA, but not PKC, and ERK pathways.

### Effects of SL327 or HA-1004 on TH phosphorylation at Ser31

**Figure 7:**
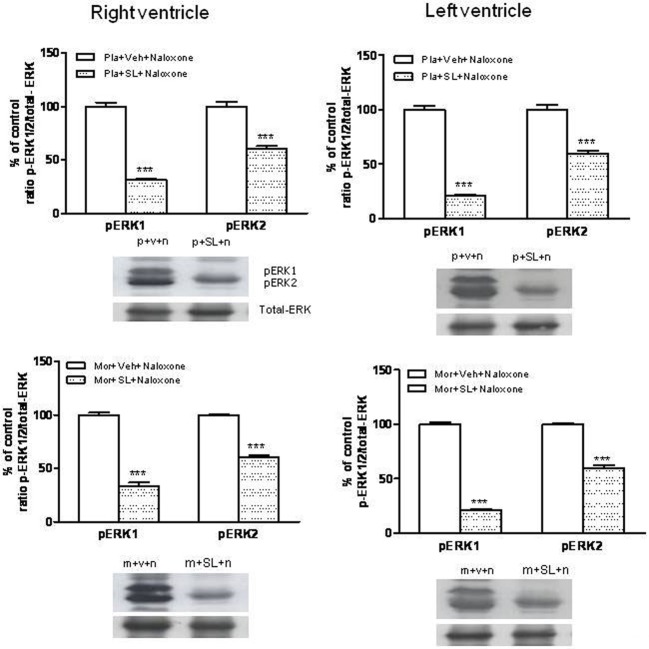
**Inmunoblots of ERK1/2 in the right and left ventricles isolated from placebo (pla,p)- or morphine (mor, m)-dependent rats after subcutaneous administration of naloxone (n) in rats treated with SL327 (SL, 100 mg/kg, i.p.) or vehicle (veh, v), 1 h before naloxone injection**. Phospho (p)ERK1 and pERK2 immunoreactive bands were measured, normalized to the background values, and expressed as percentages of controls. Data correspond to mean ± s.e.m. (*n* = 4). ^***^p < 0.001 vs. its control group. Bottom panels: representative bands from autoradiograms at the known apparent molecular weight for p-ERK1 or p-ERK2.

As previously described, TH phosphorylation at Ser31 is dependent on extracellular signal-regulated protein kinases 1 and 2 (Haycock et al., 1992). Therefore, we analyzed phospho-Ser31-TH levels in the right and left ventricle after inhibition of ERK by SL327, a drug that prevents the activation of ERK by inhibiting MEK, the upstream kinase of ERK (Atkins et al., 1998). Firstly, we determined the basal levels of phosphorylated (activated) ERK1/2 in the right and left ventricle from control and from morphine withdrawn rats pre-treated with SL327. As shown in Figure 7, phosphorylation of ERK1/2 was significantly decreased in the presence of SL327 in both controls and morphine-withdrawn animals. As SL327 effectively reduced basal levels of phospho-ERK 1/2 inmunoreactivity, we injected SL327 in control rats and in animals made dependent on morphine, 1 h before saline or naloxone and determined phospho-Ser31 TH in the right and left ventricle 90 min after the administration of the opioid antagonist.

**Figure 8:**
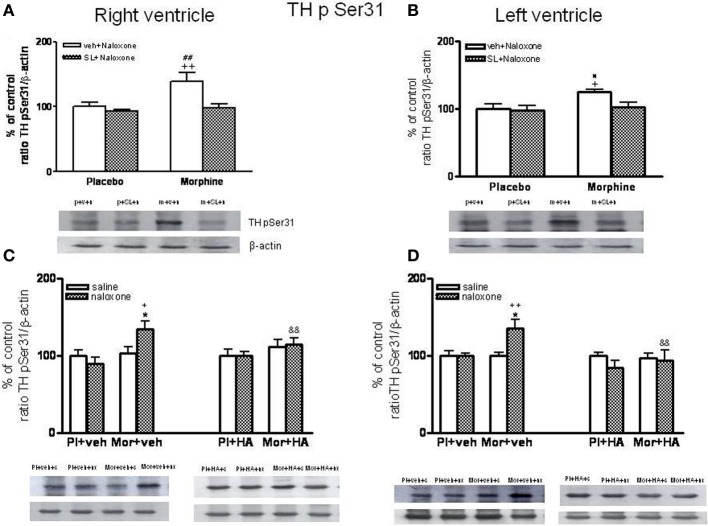
**Western blotting analysis of tyrosine hydroxylase (TH) phospho (p) Ser31 in the right ventricle and left ventricle 90 min after saline (s) or naloxone (n, nx) administration to placebo (p, Pl)- or morphine (m, Mor)-treated rats receiving vehicle (v, veh) or SL327 (SL) (A,B), veh or HA-1004 (HA) (C,D)**.The immunoreactivity corresponding to TH phospho-Ser31 is expressed respect to the control group (p+v+n or Pl+veh+saline; defined as 100% value). Data are the mean ± s.e.m. (*n* = 4–6). ^*^p < 0.05 vs. the morphine dependent group receiving saline instead of naloxone; ^+^*p* < 0.05, ^++^*p* < 0.01 vs. the group pre-treated with placebo instead of morphine injected with naloxone; ^&&^*p* < 0.001 vs. the group receiving vehicle instead of HA; ^#^*p* < 0.05, ^##^*p* < 0.01 vs. the dependent group injected with SL327. Bottom panels: representative bands from autoradiograms at the known apparent molecular weight for TH phosphorylated at Ser31.

As shown in Figures 8A,B, phospho Ser31 TH levels decreased in the right and left ventricle of morphine-dependent rats injected with SL327 before naloxone, when compared to morphine-dependent rats treated with vehicle instead of SL327. As mentioned above, SL327 effectively reduces basal levels of phospho-ERK1/2 inmunoreactivity, thereby suggesting that the decrease in phosphoSer31 TH levels after treatment with SL327 is not caused by a non-specific action of the compound on MEK. Thus, these results suggest that TH phosphorylation at Ser31 following morphine withdrawal occurs downstream of ERK.

According to a previous study (Almela et al., 2008), 60 min after naloxone-precipitated morphine withdrawal, there were not changes in the levels of phospho-Ser31 TH (data not shown). However, rats chronically treated with morphine and given naloxone showed significant increases in phospho-Ser31 TH in the right and left ventricle 90 min after the opioid antagonist injection compared with the corresponding control group receiving naloxone and with the morphine dependent animals receiving saline (Figures 8C,D). To assess the contribution of PKA to the regulation of TH, we have examined TH phosphorylation at Ser31 during morphine withdrawal in animals receiving the selective inhibitor of PKA, HA-1004. Chronic infusion of HA-1004 completely blocked the ability of naloxone-precipitated morphine withdrawal to increase the levels of phospho-Ser31 TH in the right and left ventricle (Figures 8C,D). Since SL327 failed to inhibit PKA expression in the right and left ventricle, these results demonstrated that PKA activity is required for ERK-mediated TH phosphorylation at Ser31 after morphine withdrawal.

### Cardiovascular effects of naloxone-precipitated morphine withdrawal

**Figure 9:**
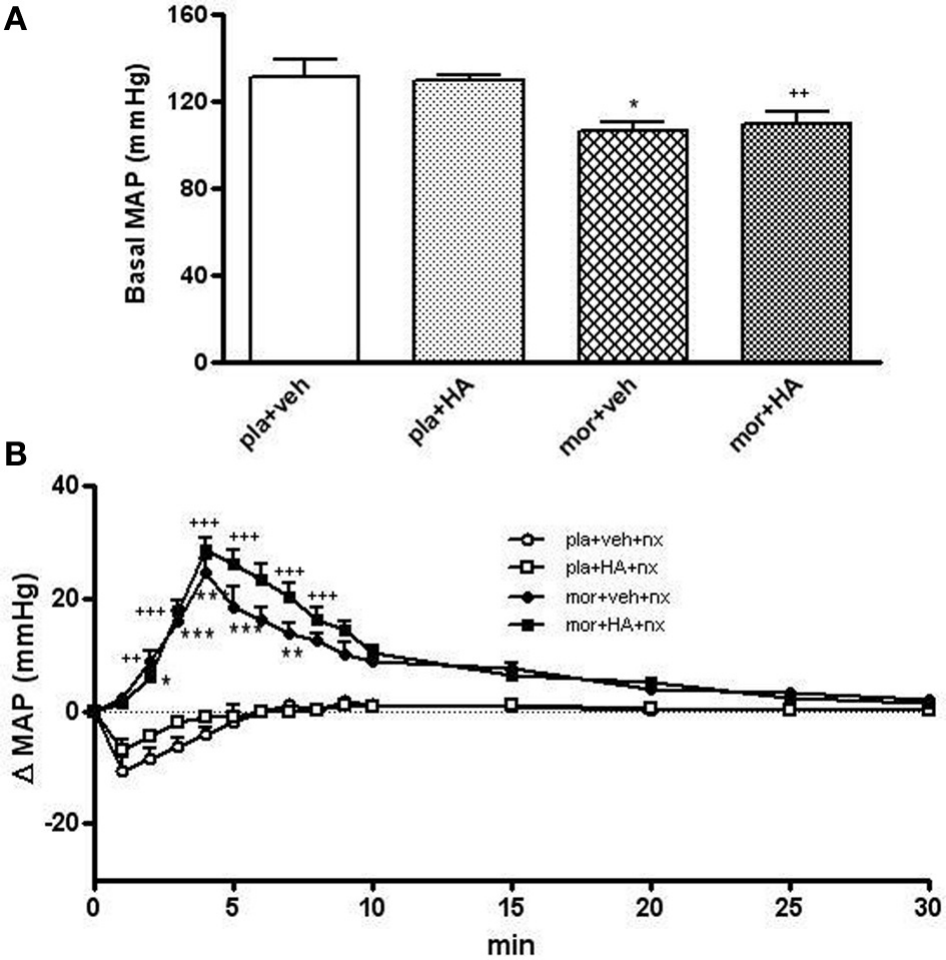
**Baseline mean arterial blood pressure (MAP, mmHg) in rats implanted with morphine (mor) or placebo (pla) pellets concomitantly with vehicle (veh) or HA-1004 (HA), just before naloxone injection (A)**. Effects of naloxone (2 mg/kg, s.c.) on changes in MAP, in rats pretreated with mor or pla concomitantly with veh or HA. Naloxone (nx) was injected at time 0 **(B)**. Data are the mean ± s.e.m. (*n* = 4–6). ^*^p < 0.05, ^**^p < 0.01, ^***^p < 0.001 vs. pla+veh; ^++^*p* < 0.01, ^+++^*p* < 0.001 vs. pla+HA.

As shown in Figures 9A,B, the pre-treatment with HA instead of vehicle did not induce significant changes in MAP. However, basal MAP was significant decreased in rats treated chronically with morphine comparing placebo group (Figure 9A). In addition, the injection of naloxone (2 mg/kg s.c.) in placebo+vehicle- or placebo+HA1004-treated rats evoked no significant changes in MAP. However, naloxone administration to morphine-dependent rats induced an immediate and significant increase in MAP (2–8 min) vs. placebo+vehicle rats injected with naloxone (Figure 9B). Similarly, the injection of naloxone in rats chronically treated with HA-1004 concomitantly with morphine induced significant changes in the MAP when compared with the morphine-dependent groups treated with vehicle instead of HA-1004 (Figure 9B).

**Figure 10:**
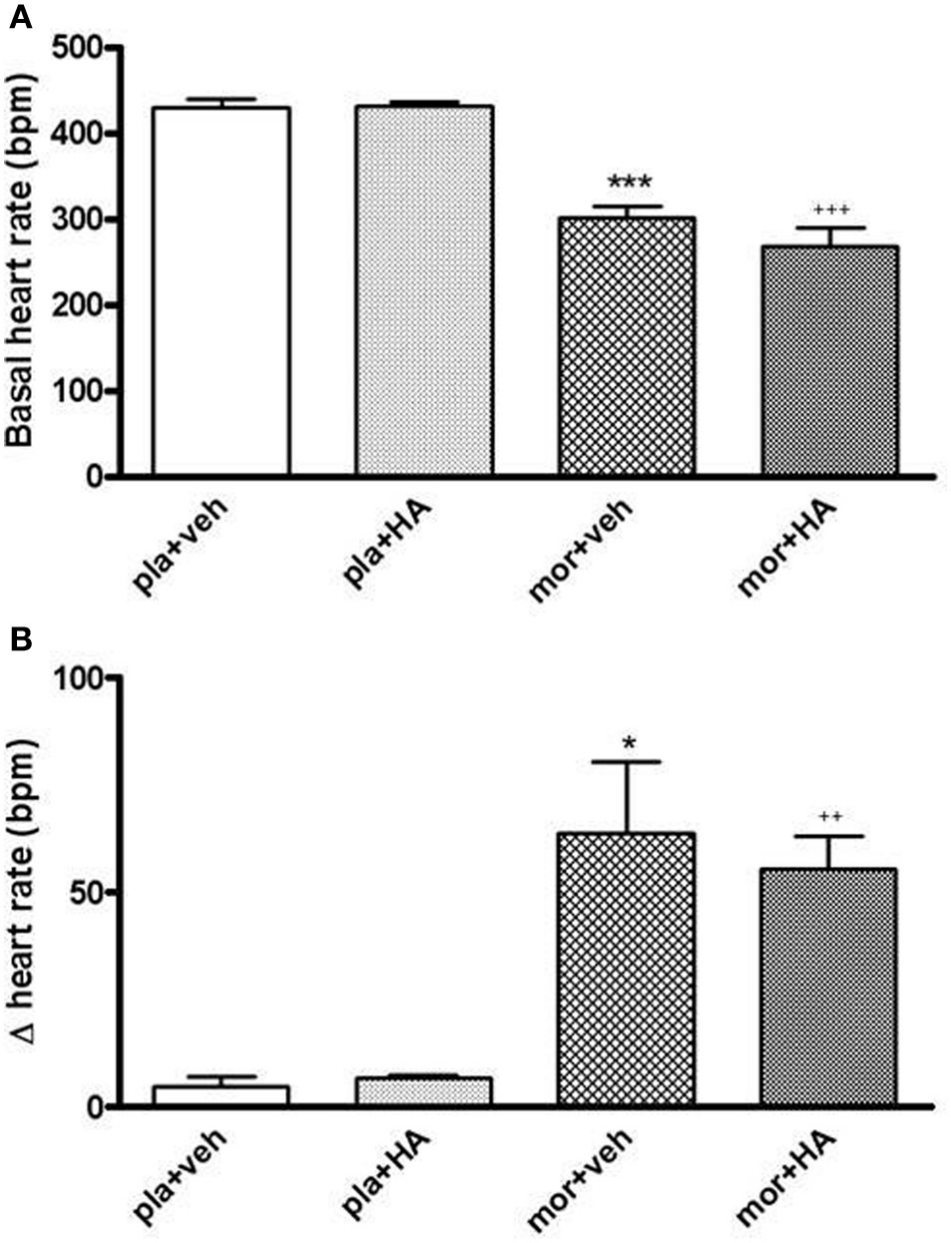
**Baseline heart rate (HR, beats per minute, bpm) in rats with morphine (mor) or placebo (pla) pellets implantation concomitantly with vehicle (veh) or HA-1004 (HA), just before naloxone injection (A)**. Changes in HR 20 min after naloxone (2 mg/kg, s.c.) injection, in rats pre-treated with mor or pla concomitantly with veh or HA **(B)**. Data are the mean ± s.e.m. (*n* = 4–6). ^*^p < 0.05, ^***^p < 0.001 vs. pla+veh; ^++^*p* < 0.01, ^+++^*p* < 0.001 vs. pla+HA.

The morphine chronic treatment also decreased the basal HR vs. placebo treatment. The rats pre-treated with placebo pellets did not show changes in HR (Figure 10A). However, 5 min after naloxone injection, we found a significant enhancement of HR in rats treated chronically with morphine. The top peak-time effect was 10 min later naloxone injection although HR remained elevated for 120 min in the morphine dependent groups (Figure 10B).

### Localization of phospho-ERK1/2 by immunohistochemistry

**Figure 11:**
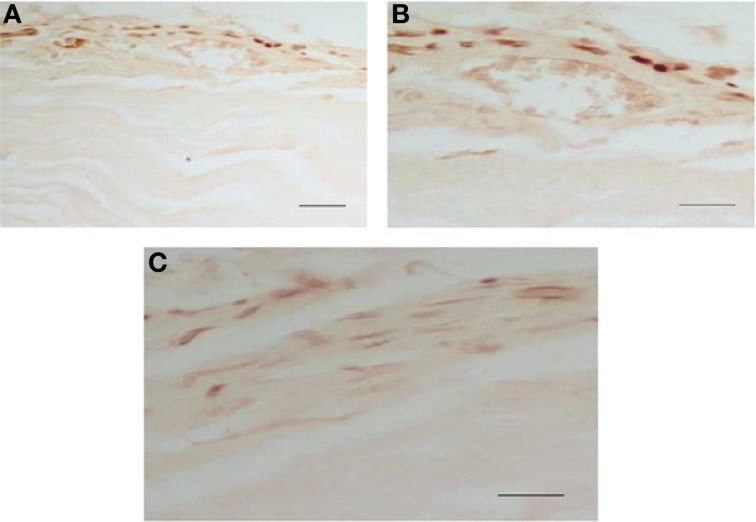
**Morphine withdrawal activates ERK1/2 in the right and left ventricle. Rats were rendered dependent on morphine during 7 days and, on day 8, were injected with saline or naloxone (subcutaneously)**. Controls groups received placebo pellets at the same time schedule and on day 8 were given with saline or naloxone. 90 min after injections, rats were perfused and the right and left ventricle were processed for phospho (p)ERK1/2 immunohistochemistry. Photographs show the immunohistochemical detection of pERK1/2 in the left ventricular wall **(A–C)**. These results are representative of four independent experiments. Normaski interference optics. Scale bar 30 mm **(A)**, 20 mm **(B,C)**. ERK, extracellular signal regulated kinase.

To obtain more information concerning the cellular localization of the phospho-ERKs in the heart, we studied the distribution of these proteins by immunohistochemical procedures using the same phospho-ERK1/2 antibody. Rats were sacrificed 90 min after saline or naloxone injection. As shown in Figure 11, high levels of phospho-ERK immunoreactivity were observed in the right and left ventricle after naloxone administration to morphine-dependent rats. The immunolabeling was mainly present in cytoplasmic compartments, suggesting a local activation of the protein. A nuclear staining was also observed in some myocytes, supporting the idea of a possible translocation of activated ERK proteins into the nucleus. By contrast, there was no staining in the right and left ventricle from control rats given naloxone or dependent rats injected with saline. These immunohistochemistry results are consistent with western blot analysis showed in the present study.

## Discussion

Our results demonstrated: (i) Morphine withdrawal activates ERK1/2 and phosphorylated tyrosine hydroxylase (TH) at Ser31 in the right and left ventricle; (ii) HA-1004 blocks the phosphorylation of ERK 1/2 and TH; and (iii) chronic morphine treatment decreases baseline cardiovascular parameters (MAP and HR). According to our finding, previous studies demonstrated that chronic μ-opiod receptor stimulation decreases muscle sympathetic nerve activity (Kienbaum et al., 2001, 2002), NA plasma concentration (Kienbaum et al., 2001), and dopamine turnover in the heart (Rabadán et al., 1997) and attenuates both contractile dysfunction and development of myocardial infarction (Gross et al., 2007; Peart et al., 2008). However, μ-opioid receptor blockade by naloxone in patients with chronic opioid abuse or in morphine dependent rats unmasks these effects, resulting in markedly increased muscle sympathetic nerve activity, NA plasma concentrations (Kienbaum et al., 2001; Peart and Gross, 2006), NA and dopamine turnover (Pugsley, 2002; Almela et al., 2008), total TH expression (Almela et al., 2008) and an increase in MAP and HR (Almela et al., 2011; Shanazari et al., 2011), two objective and accurate measurable signs of opioid withdrawal in man. Altogether, these results suggest that an up-regulation of TH would facilitate the capacity of noradrenergic neurons to synthesize NA, which could contribute to the increase in NA turnover and in the hemodynamic changes observed in the heart during morphine dependence.

Previous results from our laboratory demonstrated the capability of the selective PKA inhibitor HA-1004 for attenuating the increase of NA turnover, the total TH levels (Almela et al., 2007b) and the phosphorylation of TH (Almela et al., 2008) in morphine treated rats. However, present results show that HA-1004 failed to affect significantly MAP or HR during withdrawal. Because the cardiovascular response induced by morphine withdrawal was not altered by the PKA inhibitor, dissociation could be made between the physiological and cellular/molecular responses to naloxone-precipitated morphine withdrawal. Hence, the data of the present study suggest that the cardiovascular response and the biochemical changes which occur during morphine withdrawal could be mediated by different pathways and mechanisms.

Many pathways involved in morphine dependence are subject to feedback mechanisms that can either amplify or suppress their own signaling and there is considerable signaling from one pathway to another, a phenomenon known as crosstalk. Consequently, cell organized responses to get adapt to specific environmental conditions are the result of the sum of the intensity and duration of signals from several pathways and how they interact with each other. Since there is no evidence that PKA activation is required for stimulation of MAPK induced by morphine withdrawal in the heart (Almela et al., 2007a), this study evaluates the crosstalk between cAMP/PKA and ERKs and subsequently phosphorylation of TH at Ser31 in morphine-dependent rats. These two ancient and conserved signaling pathways are involved in many brain disorders, including drug addiction (Ren et al., 2004).

Although several signaling pathways can mediate activity-dependent phosphorylation of TH, the only PK reported to phosphorylate TH at Ser31 *in vitro* was ERK (Haycock et al., 1992; Lindgren et al., 2002). *In situ* phosphorylation of TH at Ser31 increases TH activity and catecholamine synthesis (Dunkley et al., 2004). Given that TH is phosphorylated on a specific serine residue (Ser31) by ERK, it is possible that activation of ERK1/2 in the heart provides a way in which TH is regulated under morphine dependence. In agreement with previous studies (Almela et al., 2008), we found that treatment with 100 mg/kg of SL327, a dose that selectively blocks MEK (Atkins et al., 1998; Pozzi et al., 2003), decreased the morphine withdrawal stimulation of Ser31 phosphorylation in the right and left ventricle. These data suggest that morphine withdrawal induced an activation of ERKs, which resulted in enhancement of TH phosphorylation at Ser31.

According to previous results from our laboratory obtaining by western blot and inmunohistochemistry (Almela et al., 2007a), present data shows that naloxone- induced morphine withdrawal increases phosphorylated ERK1/2 in the heart, indicating that this treatment enhances ERKs activity. In the present study, we utilized the PKA or the PKC inhibitor to check the involvement of these pathways in the activation of ERKs during morphine withdrawal. Present results demonstrate that HA-1004 antagonized the increase in ERK1/2 observed during morphine withdrawal. These data indicate that PKA signaling pathway modulates the increased levels of ERK1/2 observed during morphine withdrawal and suggest a crosstalk between PKA and ERK in the heart that could mediate the adaptive changes observed after naloxone injection to morphine dependent rats. However, the activation of ERK1/2 observed in the present study is independent of PKC pathway. According to this result, it has been indicated that there is no direct interaction between PKC and MAPK pathways mediating the effect of endothelin-1 on Glut1 transcription (Kao and Fong, 2008).

Although the mechanism of crosstalk between PKA and ERK pathways has not yet been clarified, it is possible that PKA pathway facilitates MEK1/2 which activates the ERK1/2 pathway (Stork and Schmitt, 2002; Obama et al., 2007). The activated ERK pathway increases the phosphorylation of proteins related to morphine dependence, including TH.

Targeting phosphorylated TH at Ser31 using specific antibodies, in the present study we have shown that HA-1004 blocked the increase in the level of TH phosphorylation at Ser31 induced after naloxone injection to morphine dependent rats in the right and left ventricle. These data suggest that crosstalk between PKA and ERK pathways is a key mediator design necessary to regulate the Ser31 phosphorylation of TH. In conclusion, our results indicate that TH phosphorylation at Ser 31 is regulated by ERK and PKA pathways through crosstalk mechanisms.

It has been described that tyrosine kinase receptor can use proximal located G-protein/GPCR signaling components in an integrated manner to induce activation of key regulatory pathways linked to different cellular processes (see Waters et al., 2004 for review).

It is clear from this study that PKA is implicated in the ERK activation; the activated ERK pathway increases the phosphorylation of proteins related to morphine dependence, including TH. Our data suggest that cross-talk between PKA and ERK pathways is a key regulatory design necessary to regulate the phosphorylation of TH. These findings should improve our understanding of the mechanisms involved in the cardiac adaptive changes observed during morphine withdrawal. In addition, present finding suggest that the activation of TH could be a factor that would contribute to cardiovascular alteration, such as arrhythmias, observed in heroin addicts (Nerantzis et al., 2011) and could be useful for future treatment strategies focused on addictive processes.

## Author contributions

All authors contributed equally in the preparation of this manuscript.

### Conflict of interest statement

The authors declare that the research was conducted in the absence of any commercial or financial relationships that could be construed as a potential conflict of interest.

## References

[B1] Almela P. Martínez-Laorden E. Atucha N. M. Milanés M. V. Laorden M. L. (2011). Naloxone-precipitated morphine withdrawal evokes phosphorylation of heat shock protein 27 in rat heart through extracellular signal-regulated kinase. J. Mol. Cell. Cardiol. 51, 129–139 10.1016/j.yjmcc.2011.04.002 21530534

[B2] Almela P. Milanés M. V. Laorden M. L. (2007a). Activation of ERK signalling pathway contributes to the adaptive changes in rat hearts during naloxone-induced morphine withdrawal. Br. J. Pharmacol. 151, 787–797 10.1038/sj.bjp.0707301 17549049 PMC2014132

[B3] Almela P. Cerezo M. González-Cuello A. Milanés M. V. Laorden M. L. (2007b). Differential involvement of 3,′5′-cyclic adenosine monophosphate-dependent protein kinase in regulaiton of Fos and tyrosine hydroxylase expression in the heart after naloxone induced morphine withdrawal. Naunyn Schmiedebergs Arch. Pharmacol. 374, 293–303 10.1007/s00210-006-0120-z 17216288

[B4] Almela P. Milanés M. V. Laorden M. L. (2008). The PKs PKA and ERK 1/2 are involved in phosphorylation of TH at Serine 40 and 31 during morphine withdrawal in rat hearts. Br. J. Pharmacol. 155, 73–83 10.1038/bjp.2008.224 18536752 PMC2527841

[B5] Aquaro G. D. Gabutti A. Meini M. Prontera C. Pasanisi E. Passino C. (2011). Silent myocardial damage in cocaine addicts. Heart 97, 2056–2062 10.1136/hrt.2011.226977 21690608

[B6] Atkins C. M. Selcher J. C. Petraitis J. J. Trzaskos J. M. Sweatt J. D. (1998). The MAPK cascade is required for mammalian associative learning. Nat. Neurosci. 1, 602–609 10.1038/2836 10196568

[B7] Basso C. Perazzolo M. Thiene G. (2011). Cocaine and the heart: more than just coronary disease. Heart 97, 1995–1996 10.1136/heartjnl-2011-300736 21880648

[B8] Cerezo M. Milanés M. V. Laorden M. L. (2005). Alterations in protein kinase A and different protein kinase C isoforms in the heart during morphine withdrawal. Eur. J. Pharmacol. 522, 9–19 10.1016/j.ejphar.2005.08.025 16202991

[B9] Childers S. R. (1991). Opioid receptor-coupled second messengers systems. Life Sci. 48, 1991–2003 10.1016/0024-3205(91)90154-4 1851914

[B10] Dunkley P. Bobrovskaya L. Graham M. E. von Nagy-Felsobuki E. I. Dickson P. W. (2004). Tyrosine hydroxylase phosphorylation: regulation and consecuences. J. Neurochem. 91, 1025–1043 10.1111/j.1471-4159.2004.02797.x 15569247

[B11] Frenois F. Cador M. Caille S. Stinus I. Le Moine C. (2002). Neural correlates of the motivational and somatic components of naloxone-precipitated morphine withdrawal. Eur. J. Neurosci. 16, 1377–1389 10.1046/j.1460-9568.2002.02187.x 12405997

[B12] Gold L. H. Stinus I. Inturrisi C. E. Koob G. F. (1994). Prolonged tolerance, dependence and abstinence following subcutaneous morphine pellets implantation in the rat. Eur. J. Pharmacol. 253, 45–51 10.1016/0014-2999(94)90755-2 8013548

[B13] González-Cuello A. Milanés M. V. Castells M. T. Laorden M. L. (2004). Activation of c-Fos expression in the heart after morphine but not U-50,488H withdrawal. Br. J. Pharmacol. 138, 626–633 10.1038/sj.bjp.0705093 12598416 PMC1573701

[B14] Gross E. R. Hsu A. K. Gross G. J. (2007). GSβ inhibition and K_*ATP*_ channel opening mediate acute opioid-induced cardioprotection at reperfusion. Basic Res. Cardiol. 102, 341–349 10.1007/s00395-007-0651-6 17450314

[B15] Haycock J. W. Ahn N. G. Cobbe M. H. Krebs E. G. (1992). ERK 1 and ERK 2, two microtubule-associated protein 2 kinases, mediate the phosphorylation of tyrosine hydroxylase at serine-31 in situ. Proc. Natl. Acad. Sci. U.S.A. 89, 2365–2369 10.1073/pnas.89.6.2365 1347949 PMC48658

[B16] Hidaka H. Imagaki M. Kawamoto S. Sasaki V. (1984). Isoquinolinesulfonamides, novel and potent inhibitors of cyclic nucleotide dependent protein kinase and protein kinase C. Biochemistry 23, 5036–5041 10.1021/bi00316a032 6238627

[B17] Hussain M. Drago G. A. Bhogal M. Colyer J. Orchard C. H. (1999). Effects of the protein kinase A inhibitor H-89 on Ca2+ regulation in isolated ferret ventricular myocytes. Pflugers Arch. 437, 529–537 10.1007/s004240050814 10089565

[B18] Impey S. Obrietan K. Wong S. T. Poser S. Yano S. Wayman G. (1998). Crosstalk between ERK and PKA is required for Ca2+stimulation of CREB-dependent transcription and ERK nuclear translocation. Neuron 21, 869–883 10.1016/S0896-6273(00)80602-9 9808472

[B19] Jiang X. Shi E. Nakajima Y. Sato S. (2006). COX-2 mediates morphine-induced delayed cardioprotection via an iNOS-dependent mechanism. Life Sci. 78, 2543–2549 10.1016/j.lfs.2005.10.032 16325209

[B20] Kamp T. J. Hell J.W. (2000). Regulation of cardiac L-type calcium channels by protein kinase A and protein kinase C. Circ. Res. 87, 1095–1102 10.1161/01.RES.87.12.1095 11110765

[B21] Kao Y.-S. Fong J. C. (2008). Endothelin-1 induction of Glut1 transcription in 3T-L1 adipocytes involves distinct PKCε- and p42/44 MAPK-dependent pathways. Biochem. Biophys. Acta 1780, 154–159 10.1016/j.bbagen.2007.11.013 18154738

[B22] Kienbaum P. Heuter T. Michel M. C. Scherbaum N. Gastpar M. Peters J. (2001). Chronic μ-opioid receptor stimulation in humans decreases muscle sympathetic nerve activity. Circulation 103, 850–855 10.1161/01.CIR.103.6.850 11171794

[B23] Kienbaum P. Heuter T. Scherbaum N. Gastpar M. Peters J. (2002). Chronic μ-opioid receptor stimulation alters cardiovascular regulation in humans: differential effects on muscle sympathetic and heart rate responses to arterial hypotension. J. Cardiovasc. Pharmacol. 40, 363–369 10.1097/00005344-200209000-00005 12198322

[B24] Kobayashi E. Nakano H. Morimoto M. Tamaoki T. (1989). Calphostin C (UCN), a novel microbial compound, is a highly potent and specific inhibitor of protein kinase C. Biochem. Biophys. Res. Commun. 159, 548–553 10.1016/0006-291X(89)90028-4 2467670

[B25] Laorden M. L. Fuertes G. González-Cuello A. Milanés M. V. (2000). Changes in catecholaminergic pathways innervating paraventricular nucleus and pituitary-adrenal axis response during morphine dependence: implication of A1 and A2 adrenoceptors. J. Pharmacol. Exp. Ther. 293, 578–584 10773031

[B26] Lindgren N. Goiny M. Herrera-Marschitz M. Haycock J. W. Hökfelt T. Fisone G. (2002). Activation of extracellular signal-regulated kinases 1 and 2 by depolarization stimulates tyrosine hydroxylase phosphorylation and dopamine synthesis in rat brain. Eur. J. Neuosci. 15, 769–773 10.1046/j.1460-9568.2002.01901.x 11886455

[B27] Michel M. C. Li Y. Heusch G. (2001). Mitogen-activated protein kinases in the heart. Naunyn Schmiedebergs Arch. Pharmacol. 363, 245–266 10.1007/s002100000363 11284439

[B28] Milanés M. V. Fuente T. Laorden M. L. (2000). Catecholaminergic activity and 3′,5′-cyclic adenosine monophosphate levels in heart right ventricle after naloxone induced withdrawal. Naunyn Schmiedebergs Arch. Pharmacol. 361, 61–66 10.1007/s002109900165 10651148

[B29] Milanés M. V. Fuente T. Marín M. T. Laorden M. L. (1999). Catecholaminergic activity and 3′,5′-cyclic adenosine monophosphate concentrations in the right ventricle after acute and chronic morphine administration in the rat. Br. J. Anaesth. 83, 803–815 10.1093/bja/83.5.784 10690144

[B30] Nerantzis C. E. Koulouris S. N. Marianou S. K. Pastromas S. C. Koutsaftis P. N. Agapitos E. B. (2011). Histologic findings of the sinus node and the perinodal area in street heroin addicts, victims of sudden unexpected death. J. Forensic Sci. 56, 645–648 10.1111/j.1556-4029.2011.01717.x 21361943

[B31] Nestler E. J. Aghajanian C. K. (1997). Molecular and cellular basis of addiction. Science 278, 58–63 10.1126/science.278.5335.58 9311927

[B32] Obama Y. Horgan A. Sork J. S. (2007). The requirement of Ras and Rap1 for the activation of ERKs by cAMP, PACAP, and KCL in cerebellar granule cells. J. Neurochem. 101, 470–482 10.1111/j.1471-4159.2006.04390.x 17254020

[B33] Peart J. N. Gross E. R. Reichelt M. E. Hsu A. Headrick J. P. Gross G. J. (2008). Activation of kappa-opioid receptors at reperfusion affords cardioprotection in both rat and mouse hearts. Basic Res. Cardiol. 103, 454–463 10.1007/s00395-008-0726-z 18500486

[B34] Peart J. N. Gross G. J. (2006). Cardioprotective effects of acute and chronic opioid treatment are mediated via different signalling pathways. Am. J. Physiol. Heart Circ. Physiol. 291, H1746–H1753 10.1152/ajpheart.00233.2006 16731654

[B35] Pozzi L. Häkansson K. Usiello A. Borgkvist A. Lindskog M. Greengard P. (2003). Opposite regulation by typical and atypical anti-psychotics of ERK1/2, CREB and Elk-1 phosphorylation in mouse dorsal striatum. J. Neurochem. 86, 451–459 10.1046/j.1471-4159.2003.01851.x 12871586

[B36] Pugsley M. K. (2002). The diverse molecular mechanism responsible for the actions of opioids on the cardiovascular system. Pharmacol. Ther. 93, 51–57 10.1016/S0163-7258(02)00165-1 11916541

[B37] Rabadán J. V. Milanés M. V. Laorden M. L. (1997). Effects of chronic morphine treatment on catecholamines content and mechanical response in the rat heart. J. Pharmacol. Exp. Ther. 280, 32–37 8996178

[B38] Rabadán J. V. Milanés M. V. Laorden M. L. (1998). Changes in right atrial catecholamine content in naïve rats and after naloxone-induced withdrawal. Br. J. Anaesth. 80, 354–359 10.1093/bja/80.3.354 9623438

[B39] Ren X. Noda Y. Mamiya T. Nagai T. Nabeshima T. A. (2004). Neuroactive steroid, dehydroepiandreosterone sulfate, prevents the development of morphine dependence and tolerance via c-Fos expression linked to the extracellular signal-regulated protein kinase. Behav. Brain Res. 152, 243–250 10.1016/j.bbr.2003.10.013 15196791

[B40] Sengupta N. Vinod P. K. Venkatesh K. V. (2007). Crosstalk between cAMP/PKA and MAP kinase pathways is a key regulatory design necessary to regulate FLO11 expression. Biophys. Chem. 125, 59–71 10.1016/j.bpc.2006.06.012 16863676

[B41] Shanazari A. A. P. Aslani Z. Ramshini E. Alaei H. (2011). Acute and chronic effects of morphine on cardiovascular system and the baroreflexes sensitivity during severe increase in blood pressure in rats. ARYA Atheroscler. 7, 111–117 22577457 PMC3347855

[B42] Sindreu C. B. Scheiner Z. S. Storm D. R. (2007). Ca2+-stimulated adenylyl cyclases regulate ERK-dependent activation of MSK1 during fear conditioning. Neuron 53, 79–89 10.1016/j.neuron.2006.11.024 17196532 PMC1858648

[B43] Stork P. Schmitt J. (2002). Crosstalk between cAMP and MAP kinase signalling in the regulation of cell proliferation. Trends Cell Biol. 12, 258–266 10.1016/S0962-8924(02)02294-8 12074885

[B44] Sugden P. H. Bogoyevitch M. A. (1995). Intracellular signalling through protein kinases in the heart. Cardiovasc. Res. 30, 478–492 8574996

[B45] Ueda H. (2004). Locus-specific involvements of anti-opioid systems in morphine tolerance and dependence. Ann. N.Y. Acad. Sci. 1025, 376–382 10.1196/annals.1307.046 15542739

[B46] Villalba M. Bockaert J. Journot L. (1997). Pituitary adenylate cyclase-activating polypeptide (PACAP-38) protects cerebellar granule neurons from apoptosis by activating the mitogen-activated protein kinase (MAP kinase) pathway. J. Neurosci. Res. 17, 83–90 8987738 10.1523/JNEUROSCI.17-01-00083.1997 PMC6793681

[B47] Vossler M. Yao H. Pan M. Rim C. Stork P. (1997). cAMP activates MAP kinase and Elk-1 trough a B-Raf and Rap-1-dependent pathway. Cell 89, 73–82 10.1016/S0092-8674(00)80184-1 9094716

[B48] Waters C. Pyne S. Pyne N. J. (2004). The role of G-protein coupled receptors and associated proteins in receptor tyrosine kinase signal transduction. Semin. Cell Dev. Biol. 15, 309–323 10.1016/j.semcdb.2003.12.020 15125894

[B49] Wiechelman K. J. Braund R. D. Fitzpatrick J. D. (1988). Investigation of the bicincinomic acid protein assay: identification of the groups responsible for colour formation. Anal. Biochem. 175, 231–237 10.1016/0003-2697(88)90383-1 3245570

[B50] Xu J. Tian W. Ma X. Guo J. Shi Q. Jin Y. (2011). The molecular mechanism underlying morphine-induced Akt activation: roles of protein phosphatases and reactive oxygen species. Cell Biochem. Biophys. 61, 303–311 10.1007/s12013-011-9213-5 21626435

